# Automated detection and quantification of brain metastases on clinical MRI data using artificial neural networks

**DOI:** 10.1093/noajnl/vdac138

**Published:** 2022-08-23

**Authors:** Irada Pflüger, Tassilo Wald, Fabian Isensee, Marianne Schell, Hagen Meredig, Kai Schlamp, Denise Bernhardt, Gianluca Brugnara, Claus Peter Heußel, Juergen Debus, Wolfgang Wick, Martin Bendszus, Klaus H Maier-Hein, Philipp Vollmuth

**Affiliations:** Department of Neuroradiology, Heidelberg University Hospital, Heidelberg, Germany; Medical Image Computing, German Cancer Research Center (DKFZ), Heidelberg, Germany; Medical Image Computing, German Cancer Research Center (DKFZ), Heidelberg, Germany; Department of Neuroradiology, Heidelberg University Hospital, Heidelberg, Germany; Department of Neuroradiology, Heidelberg University Hospital, Heidelberg, Germany; Department of Diagnostic and Interventional Radiology with Nuclear Medicine, Clinic for Thoracic Diseases (Thoraxklinik), Heidelberg University Hospital, Heidelberg, Germany; Department of Radiation Oncology, Klinikum rechts der Isar, Technical University Munich, Munich, Germany; Department of Neuroradiology, Heidelberg University Hospital, Heidelberg, Germany; Department of Diagnostic and Interventional Radiology with Nuclear Medicine, Clinic for Thoracic Diseases (Thoraxklinik), Heidelberg University Hospital, Heidelberg, Germany; Member of the Cerman Center for Lung Research (DZL), Translational Lung Research Center (TLRC), Heidelberg, Germany; Department of Radiation Oncology, Heidelberg University Hospital, Heidelberg, Germany; Heidelberg Institute for Radiation Oncology (HIRO), Heidelberg University Hospital, Heidelberg, Germany; German Cancer Consotium (DKTK), National Center for Tumor Diseases (NCT), German Cancer Research Center (DKFZ), Heidelberg, Germany; Clinical Cooperation Unit Radiation Oncology, German Cancer Research Center (DKFZ), Heidelberg, Germany; Neurology Clinic, Heidelberg University Hospital, Heidelberg, Germany; Clinical Cooperation Unit Neurooncology, German Cancer Consortium (DKTK), German Cancer Research Center (DKFZ), Heidelberg, Germany; Department of Neuroradiology, Heidelberg University Hospital, Heidelberg, Germany; Medical Image Computing, German Cancer Research Center (DKFZ), Heidelberg, Germany; Department of Neuroradiology, Heidelberg University Hospital, Heidelberg, Germany

**Keywords:** artificial intelligence, artificial neural network, brain metastasis, magnetic resonance imaging, neuro-Oncology

## Abstract

**Background:**

Reliable detection and precise volumetric quantification of brain metastases (BM) on MRI are essential for guiding treatment decisions. Here we evaluate the potential of artificial neural networks (ANN) for automated detection and quantification of BM.

**Methods:**

A consecutive series of 308 patients with BM was used for developing an ANN (with a 4:1 split for training/testing) for automated volumetric assessment of contrast-enhancing tumors (CE) and non-enhancing FLAIR signal abnormality including edema (NEE). An independent consecutive series of 30 patients was used for external testing. Performance was assessed case-wise for CE and NEE and lesion-wise for CE using the case-wise/lesion-wise DICE-coefficient (C/L-DICE), positive predictive value (L-PPV) and sensitivity (C/L-Sensitivity).

**Results:**

The performance of detecting CE lesions on the validation dataset was not significantly affected when evaluating different volumetric thresholds (0.001–0.2 cm^3^; *P* = .2028). The median L-DICE and median C-DICE for CE lesions were 0.78 (IQR = 0.6–0.91) and 0.90 (IQR = 0.85–0.94) in the institutional as well as 0.79 (IQR = 0.67–0.82) and 0.84 (IQR = 0.76–0.89) in the external test dataset. The corresponding median L-Sensitivity and median L-PPV were 0.81 (IQR = 0.63–0.92) and 0.79 (IQR = 0.63–0.93) in the institutional test dataset, as compared to 0.85 (IQR = 0.76–0.94) and 0.76 (IQR = 0.68–0.88) in the external test dataset. The median C-DICE for NEE was 0.96 (IQR = 0.92–0.97) in the institutional test dataset as compared to 0.85 (IQR = 0.72–0.91) in the external test dataset.

**Conclusion:**

The developed ANN-based algorithm (publicly available at www.github.com/NeuroAI-HD/HD-BM) allows reliable detection and precise volumetric quantification of CE and NEE compartments in patients with BM.

**Key Points**

- Assisting practitioners to overcome limitations of manual assessment of tumor burden.
- High performance on heterogeneous MRI data and brain metastases lesions of small sizes.
- Publicly available artificial neural network based brain metastases segmentation algorithm.

**Importance of the Study**

Treatment efficacy according to the Response Assessment in Neuro-Oncology Brain Metastases (RANO-BM) is highly dependent on the tumor growth dynamic, which relies on accurately detecting brain metastases (BM) instances and estimating their volumetric extent correctly. Due to the difficulties and time-intensive nature of this task artificial neural networks (ANN) based methods have been proposed to automate this process, firstly for brain tumors and recently also for BM. This study expands on previous work to more challenging clinical settings with data from varying stages of treatment and improves performance for small BM instances.

About 25–45% of all patients with extracranial, malignant primary tumors develop brain metastases (BM).^1,2^ Despite multimodal treatments the life expectancy of the patients who develop BM is still poor, with median survival of 2–18 months.^2,3^ In this context, the determination of the exact endpoints of the treatment effectiveness plays a key role in neuro-oncology. One of the essential criteria for the precise assessment of the efficiency of a new therapy for brain tumors is the growth dynamics determined by magnetic resonance imaging (MRI) based mainly on manual measurements of target lesions according to the Response Assessment in Neuro-Oncology Brain Metastases (RANO-BM) criteria.^4^ Although manual measurements of the largest diameter as prescribed by the RANO-BM criteria allow easy and widespread adoption in clinical practice, previous studies have shown that volumetric measurement may provide a more reliable and accurate metric.^5–7^ The clinical potential of volumetric measurements and the possibility of automating this laborious analysis through artificial neural networks (ANN) has primarily been demonstrated in the setting of primary brain tumors,^8–16^ whereas only a limited number of studies have investigated these approaches in the setting of BM.^17–28^ Prior studies that have evaluated the performance of ANN for the detection and/or segmentation of BM have shown promising results but have also been limited by a relative high number of false positive (FP) results (ranging from 1.5 to 20 per case)^17,20,24–26^ and relatively poor performance in detecting smaller BM (high number of false negative with reported F1-scores in the range of 0.76–0.85).^19,21,23^ Moreover, available studies so far only focus on segmenting the contrast-enhancing tumors (CE) lesion of BM whereas they do not quantify the surrounding non-enhancing FLAIR signal abnormality/ edema (NEE) which may be particularly important in the context of evaluating post-treatment changes during follow-up of BM.

Here, we evaluated the potential of a state-of-the-art ANN-based on the self-configuring nnU-Net method^29^ for automated detection and quantification of CE lesions and NEE in BM using MRI data from a large institutional dataset for training, validation and testing. We evaluated detection and segmentation performance of the developed ANN on a case- and lesion-wise basis and analyzed the dependence of these metrics on the size of BM. Moreover, we applied the ANN to an independent external dataset, thereby enabling to evaluate the generalization of the model across multisite data.

## Material and Methods

### Datasets

The retrospective analysis of imaging data was approved by the local ethics committee of the Medical Faculty of the University of Heidelberg and informed consent was waived. The following datasets were used for the present study:

### Institutional Dataset

To develop, train and test an ANN for automated interpretation of MRI data in clinical setting we collected MRI data (*n* = 308) of adult patients (mean age 61 ± 11 years; 163 female) with BM from several primary cancers, who underwent standardized MRI examination for radiation treatment planning at Heidelberg University Hospital between 04/2011 and 04/2018. We included the last MRI scan prior to the start of radiation therapy. No exclusions were made based on the primary tumor histology or time-point of MRI exam, neither initially at primary diagnosis of BM, nor early post-operatively or follow-up, with the goal of exposing the ANN to as many different appearances of BM on MRI and thus enabling it to learn a broad range of clinical scenarios. The institutional MRI dataset was divided into a training/validation and a test dataset with a ratio of 4:1. Specifically, the institutional training/validation dataset consisted of 246/308 (80%) patients and the institutional test dataset consisted of 62/308 (20%) patients.

### External Dataset

Another cohort of 30 adult patients (mean age 58 ± 11 years; 15 female) with lung cancer and at least one BM, who underwent routine MRI scans at the Heidelberg Thoracic Clinic between 06/2013 and 08/2019 was used to verify the generalisability of our developed method. This dataset consisted of MRI data at the time point of first occurrence of BM in the course of the disease.

### Image Acquisition

MRI exams of the institutional dataset were acquired with a 3 T MRI system (Magnetom Verio, Skyra or Trio TIM; Siemens Healthineers), except a single measurement of the training set, which was acquired with a 1.5-T field strength (Magnetom Avanto; Siemens Healthineers). All MRI exams of the external test dataset were acquired with a 1.5-T MRI system (Magnetom Avanto; Siemens Healthineers). MRI scans from all datasets were acquired according to an established protocol and included T1-weighted images before and after gadolinium contrast agent and FLAIR images (detailed description of acquisition parameters in the Supplement).

### Image Preprocessing

The MRI data were processed as described in Kickingereder et al.^8^ Briefly, this included deep-learning based brain extraction using HD-BET,^30^ image co-registration, and calculation of T1-subtraction maps (T1-sub). Subsequently, ground-truth segmentation of the BM was performed using ITK-SNAP (www.itksnap.org), as described in Kickingereder et al.^8^ by IP, an in-training radiologist with 5 years of experience and subsequently checked by PV a board-certified neuroradiologist with 10 years of experience. Any discrepancies were resolved through consensus discussion. Specifically, CE lesions (on the T1-sub images or in case of artifacts on T1-sub with additional support of T1-weighted post-contrast images) as well as the associated NEE (excluding the contrast-enhancing and necrotic portion of the BM, resection cavity and obvious leukoaraiosis) were selected using a region-growing segmentation algorithm.

### Artificial Neural Network

The architecture of the developed ANN (termed HD-BM) was based on the BraTS 2020 winning,^31^ self-configuring nnU-Net method,^29^ which itself is based on the U-Net,^32^ that has shown to have excellent performance in brain tumor segmentation in the context of a large-scale multi-institutional study.^8^ During training, the model receives all input modalities of each training sample and was taught to reproduce the provided reference annotation. We followed the original, state-of-the-art nnU-Net training regime closely by training an ensemble of five models on our institutional train dataset, through the means of five-fold cross-validation. This splits the dataset into five partially overlapping training and five mutually exclusive validation subsets. Consequently, each of the images contained in the institutional training data set was used for validation once, allowing us to report validation metrics for our training cohort. Both test datasets remained untouched until model development was completed. Only then was the final model configuration used to generate predictions. These predictions were subsequently used in the performance analysis. Through development of additional models we additionally investigate how only receiving the T1-weighted images after gadolinium contrast agent and FLAIR images influences the performance, which we subsequently refer to as “Slim”. A detailed description of the applied ANN architecture and discussion of the Slim configuration is available in the Supplement.

### Statistical Analysis and Evaluation Metrics

The performance of HD-BM for detecting and segmenting BM in both datasets was assessed case-wise for CE and NEE and lesion-wise for CE using the case-wise/lesion-wise DICE-coefficient (C/L-DICE), sensitivity (C/L-Sensitivity). In addition, for CE lesions we calculated the lesion-wise positive predictive value (L-PPV), sensitivity (L-Sensitivity) as well as F1-score. For volume agreement we report concordance correlation coefficient (CCC) lesion-wise for CE lesions and case-wise for NEE parts of BM. To evaluate the detection and segmentation performance between and within the respective datasets, we performed the Wilcoxon test and Spearman correlation. *P* < .05 was considered significant. The statistical analyses were performed using R version 4.0.3 (https://www.r-project.org) and Python version 3.9.7 (http://www.python.org). More information regarding the statistical analysis is provided in the Supplement.

## Results

### Size and Distribution of Brain Metastases

Table 1 provides detailed characteristics of the included patients and BM. A total of 1682 BM were segmented in the institutional training/validation dataset, 384 BM in the institutional test dataset, and 155 BM in the external test dataset. The average number of BM (CE lesions) per patient was similar between the training/validation dataset (7 ± 15) and the institutional test dataset (6 ± 11) as well as the external test dataset (5 ± 8) (*P* = .986). The average volume of individual CE lesions was similar between the training/validation dataset (1.23 ± 4.59 cm^3^) and the institutional test dataset (1.24 ± 4.46 cm^3^; *P* = .2258), whereas it was significantly smaller in the external test dataset (1.03 ± 5.17 cm^3^, *P* = .0392 on comparison with the institutional test dataset). Similarly, the average volume of NEE per case was similar between the training/validation dataset (58.61 ± 55.54 cm^3^) and the institutional test dataset (62.1 ± 52.65 cm^3^; *P* = .334), whereas it was significantly smaller in the external test dataset (36.81 ± 52.69 cm^3^; *P* = .005 in comparison with the institutional test dataset). This discrepancy in volume for the both lesion classes in test datasets might be explained due to the significantly higher number of cases with surgical resection in the institutional test dataset 20/62 (32%) as compared to the external dataset with only one case out of 30 (3%) (p=0.0019).

**Table 1.** Characteristics of the Patients Included in This Study

	Institutional dataset		External test dataset	*P* value
	Training set	Test set		
Patient [*n*]	246	62	30	–
Gender [*n*, (%)]				.530
Female	134 (54.5)	29 (46.7)	15 (50)	
Male	112 (45.5)	33 (53.3)	15 (50)	
Mean age [years (± SD)]	61 (± 11)	61 (± 12)	58 (± 12)	.454
No. of metastases (total)	1682	384	155	–
Mean no. of metastases per patient (± SD)	7 (± 15)	6 (± 11)	5 (± 8)	.986
Case-wise volumes				
CE-Lesion				.007
Mean CE-lesion volume (± SD)	8.47 cm^3^ (± 12.11)	7.81 cm^3^ (± 9.77)	5.31 cm^3^ (± 12.33)	
Median CE-lesion volume (IQR)	3.91 cm^3^ (9.4)	5.31 cm^3^ (8.63)	0.63 cm^3^ (4.38)	
NEE-Lesion				0.014
Mean NEE-lesion volume (± SD)	58.61 cm^3^ (± 55.54)	62.1 cm^3^ (± 52.65)	36.81cm^3^ (± 52.69)	
Median NEE-lesion volume (IQR)	42 cm^3^ (78.69)	49.22 cm^3^ (59.82)	10.10 cm^3^ (67.25)	
Lesion-wise volumes				.141
Mean CE-lesion volume (± SD)	1.23 cm^3^ (± 4.59)	1.24 cm^3^ (± 4.46)	1.03 cm^3^ (± 5.17)	
Median CE-lesion volume (IQR)	0.07 cm^3^ (0.33)	0.05 cm^3^ (0.29)	0.08 cm^3^ (0.33)	
Primary cancer [(*n*, (%)]				.256
Lung	97 (39.4)	27 (43.5)	30 (100)	
Breast	59 (24)	9 (14.5)	–	
Gastrointestinal	17 (6.9)	5 (8.1)	–	
Cancer of unknown primary origin	15 (6.1)	3 (4.8)	–	
Kidney	12 (4.9)	4 (6.5)	–	
Malignant melanoma	10 (4.1)	8 (12.9)	–	
Soft-tissue sarcoma	4 (1.6)	1 (1.6)	–	
Multiple primary tumors	4 (1.6)	–	–	
Prostate	3 (1.2)	–	–	
Others	25 (10.2)	5 (8.1)	–	
MRI sequence [*n*, (%)]				–
T1-w				
3D acquisition	212 (86.2)	54 (87.1)	30 (100)	
2D acquisition	34 (13.8)	8 (12.9)	–	
cT1-w	246 (100)	62 (100)	30 (100)	
FLAIR	246 (100)	62 (100)	30 (100)	
MR vendors (field strength) [*n*, (%)]				–
Siemens (1.5 T)	–	1 (1.6)	30 (100)	
Siemens (3.0 T)	246 (100)	61 (98.4)	–	

SD, standard deviation; IQR, inter-quartile range; T, Tesla; CE, contrast-enhancing tumors; NEE, non-enhancing FLAIR signal abnormality/edema.

Group differences were evaluated with chi-square test for categorical and Kruskal–Wallis test or *t* test (depending on the distribution) for continuous parameters.

The types of primary cancers were balanced between the institutional training/validation and test dataset (*P* = .256) with the most common entities being lung and breast cancer. In contrast the composition of primary cancers in the external test dataset was different and exclusively consisted of lung cancer patients, thereby reflecting the treatment focus of the Heidelberg Thoracic Clinic from which the external test dataset originated.

### Detection and Segmentation Performance of HD-BM in the Validation Dataset

**Figure 1.**
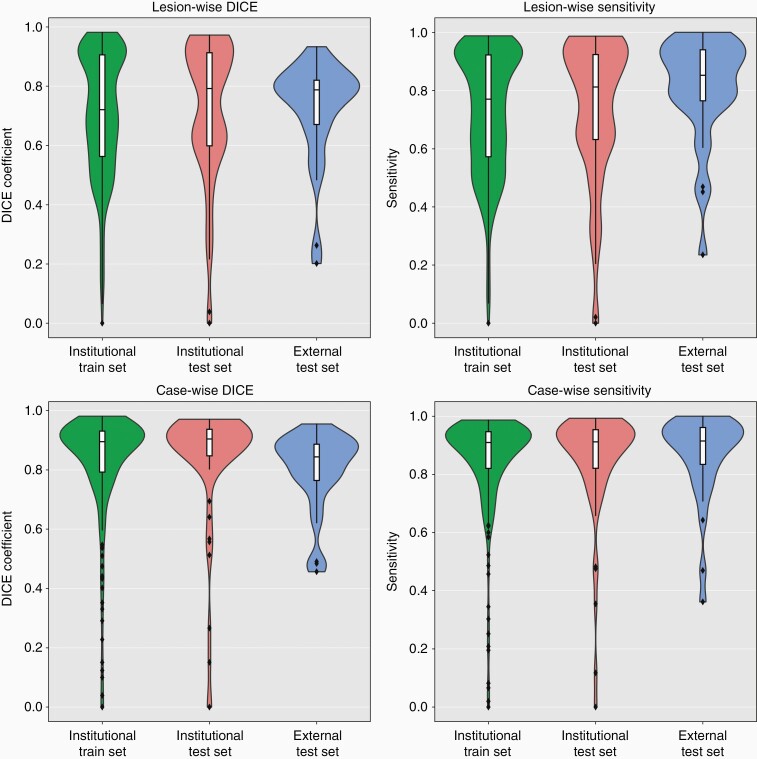
Segmentation (left column) and detection (right column) agreement between the ground-truth segmentation mask generated by the radiologist and the automatically generated segmentation masks for contrast-enhancing (CE) tumor on a per lesion level (upper row) and a per case level (lower row) within each dataset using violin charts and superimposed box plots. The colors represent each data set.

Table 2 and Figure 1 encompass detailed results on the performance of HD-BM in the validation dataset. Specifically, the case-wise sensitivity and DICE-coefficient was 0.91 (IQR = 0.82–0.95) and 0.90 (IQR = 0.79–0.93) for CE lesions as well as 0.95 (IQR = 0.87–0.98) and 0.95 (IQR = 0.88–0.97) for the NEE part of the BM.

**Table 2.** Case-wise Segmentation Quality for Contrast-enhancing Tumors (CE) and Non-enhancing FLAIR Signal Abnormality/edema (NEE) and Lesion-wise Segmentation and Detection Quality for CE Lesions for the Institutional Training Set, Institutional Test Set and External Test Set

	Institutional dataset						External dataset		
	Training set			Test set			Test set		
	Full-model	“Slim”-model	*P* value	Full-model	“Slim”-model	*P* value	Full-model	“Slim”-model	*P* value
Case-wise volumes									
CE-Lesion									
C-DICE (IQR)	0.90 (0.79–0.93)	0.87 (0.73–0.92)	< .001	0.90 (0.85–0.94)	0.89 (0.81–0.93)	< .001	0.84 (0.76–0.89)	0.83 (0.70–0.89)	.07
C-Sensitivity (IQR)	0.91 (0.82–0.95)	0.89 (0.78–0.94)	< .001	0.91 (0.82–0.95)	0.89 (0.80–0.95)	.003	0.91 (0.83–0.96)	0.89 (0.74–0.93)	< .001
NEE-Lesion									
C-DICE (IQR)	0.95 (0.88–0.97)	0.95 (0.88–0.97)	.03	0.96 (0.92–0.97)	0.96 (0.92–0.97)	.2	0.85 (0.72–0.91)	0.86 (0.72–0.91)	.01
C-Sensitivity (IQR)	0.95 (0.87–0.98)	0.95 (0.87–0.98)	.57	0.95 (0.91–0.98)	0.95 (0.91–0.98)	.5	0.91 (0.83–0.97)	0.92 (0.83–0.97)	.26
Lesion-wise volumes									
CE-Lesion									
L-DICE (IQR)	0.72 (0.56–0.90)	0.72 (0.53–0.88)	< .001	0.78 (0.60–0.91)	0.71 (0.53–0.88)	.0015	0.79 (0.67–0.82)	0.77 (0.59–0.81)	.51
L-Sensitivity (IQR)	0.77 (0.57–0.92)	0.76 (0.56–0.91)	.003	0.81 (0.63–0.92)	0.72 (0.54–0.91)	.03	0.85 (0.76–0.94)	0.81 (0.65–0.91)	.02
L-PPV (IQR)	0.82 (0.65–0.93)	0.80 (0.64–0.92)	.01	0.79 (0.63–0.93)	0.79 (0.65–0.91)	.35	0.76 (0.68–0.88)	0.79 (0.68–0.90)	.03
F1-Score (IQR)	0.94 (0.75–1.0)	0.92 (0.75–1.0)	.02	0.93 (0.80–1.0)	0.96 (0.68–1.0)	.85	1.0 (0.89–1.0)	1.0 (0.81–1.0)	.4
Mean F1-Score (SD)	0.86 (± 0.19)	0.83 (± 0.23)	.02	0.83 (± 0.24)	0.83 (± 0.24)	.85	0.90 (± 0.19)	0.89 (± 0.17)	.4

CE, contrast-enhancing tumors; NEE, non-enhancing FLAIR signal abnormality/edema; PPV, positive predictive value.

The “Slim”-Model refers to a configuration where the HD-BM is trained only with T1-weighted images after gadolinium contrast agent and FLAIR images and is discussed in detail in the supplement. All values but one are median with respective inter-quartile ranges (IQR) except one mean F1-Score with standard deviation (SD). Comparison of respective datasets on the basis of the necessary input sequences: full-model (T1-weighted images before and after gadolinium contrast agent, FLAIR images and T1-subtraction map) and “Slim”-model (T1-weighted images after gadolinium contrast agent and FLAIR images). Group differences were evaluated with Wilcoxon test.

Analysis of the performance of HD-BM for detecting and segmenting CE lesions on a lesion-wise level demonstrated an F1-score of 0.94 (IQR = 0.76–1.0), with a L-sensitivity of 0.77 (IQR = 0.57–0.92) and L-PPV of 0.82 (IQR = 0.65–0.93) resulting in a L-DICE-coefficient of 0.72 (IQR = 0.56–0.90). To evaluate if the F1-score improves when filtering predicted instances by their volume, we calculated it over a range of volume thresholds ranging 0.001–0.2 cm^3^ (Supplementary Figure S2). By filtering instances < 0.006cm^3^ the F1-score increased from mean 0.86 ± 0.19 to its maximum value of mean 0.87 ± 0.19. However, since this increase was non-significant (*P* = .203) no volumetric threshold was applied for subsequent analyses.

### Detection and Segmentation Performance of HD-BM in the Test Datasets

**Figure 2.**
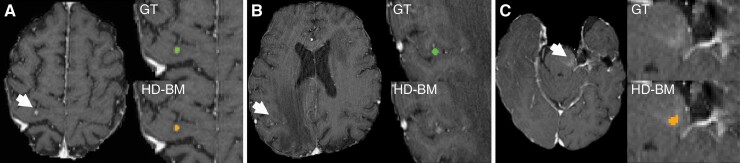
Example of true positive (A), false negative (B) and false positive (C) findings in the institutional/external dataset. Three example MRI studies with axial T1-weighted postcontrast images. (A) HD-BM (orange) shows accurate detection of BM (white arrow) in the right precentral gyrus comparable to the ground-truth (GT) segmentation (green). (B) Missed BM (green) were mostly small or associated with subtle contrast enhancement as shown here in the right parietal lobe (white arrow). (C) False positive findings (orange) were predominantly associated with vascular changes (white arrow; capillary telangiectasia).

**Figure 3.**
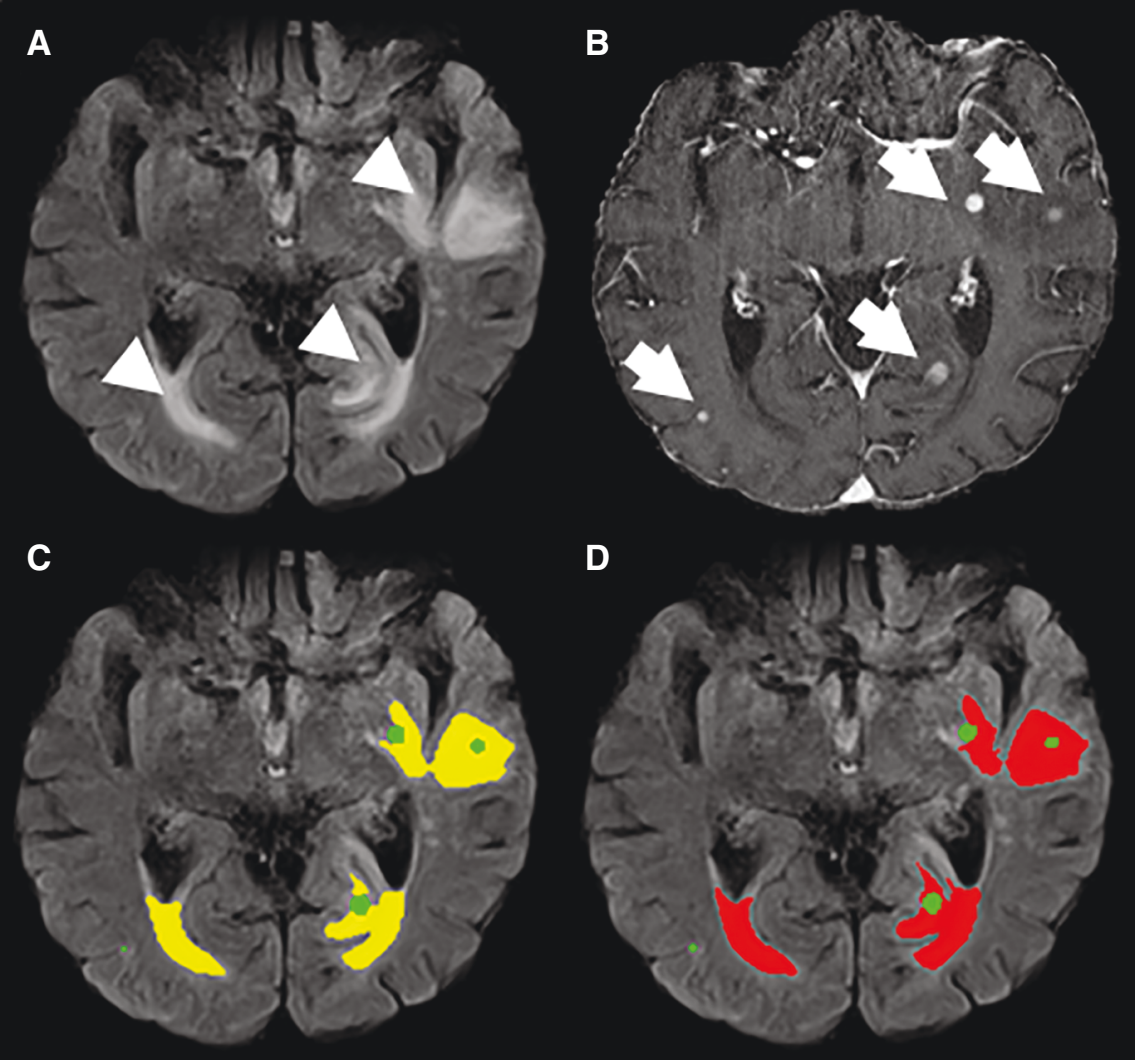
Example of an MRI study with axial FLAIR and T1-weighted post-contrast images of a 71-year-old male patient with malignant melanoma and multiple BM in the institutional dataset (B, arrows and green) and perifocal edema (A, arrowhead). Our HD-BM algorithm detects the perifocal edema accurately (C, yellow) compared to the ground-truth segmentation (D, red).

Table 2 and Figure 1 encompass detailed results on the performance of HD-BM in both institutional and external test dataset. Exemplary predicted segmentations of the distinct test set patients are shown in Figures 2 and 3. The case-wise median C-sensitivity and median C-DICE in the institutional test dataset was 0.91 (IQR = 0.82–0.95) and 0.90 (IQR = 0.85–0.94) for CE lesions as well as 0.95 (IQR = 0.91–0.98) and 0.96 (IQR = 0.92–0.97) for the NEE part of the BM. In contrast, the case-wise median C-sensitivity and median C-DICE in the external test dataset were 0.91 (IQR = 0.83–0.96) and 0.84 (IQR = 0.76–0.89) for CE lesions as well as 0.91 (IQR = 0.83–0.97) and 0.85 (IQR = 0.72–0.91) for the NEE part of BM. Comparing these metrics between the institutional and external test dataset demonstrated similar C-sensitivity (*P* = .761) but lower C-DICE (*P* = .002) of CE lesions as well as lower C-sensitivity (*P* = .018) and C-DICE (*P* < .001) of the NEE part of BM in the external test dataset. The number of FP/scan was 0.87 in the institutional and 0.20 in the external test dataset.

The volume of the individual CE lesions significantly influenced the segmentation performance (L-DICE) of individual CE lesions (Spearman’s *r* = .789 with *P* < .001 in the institutional test dataset and Spearman’s *r* = .555 with *P* < .001 in the external test dataset) (Supplementary Figure S3). Similarly, the volume of the NEE part of BM did also significantly influence the segmentation performance (C-DICE) of the NEE part of BM on a case-wise level (Spearman’s *r* = .642 with *P* < .001 in the institutional test dataset and Spearman’s *r* = .697 with *P* < .001 in the external test dataset) (Supplementary Figure S3). Consequently, the significantly lower volumes of individual CE lesions and NEE part of BM in the external test dataset as compared to the institutional test dataset likely explains the relative performance drop of HD-BM in the external test dataset.

Analysis of the performance of HD-BM for detecting CE lesions on a lesion-wise level demonstrated a median F1-score score of 0.93 (IQR = 0.80–1.0) with a median L-sensitivity of 0.81 (IQR = 0.63–0.92) and a median L-PPV of 0.79 (IQR = 0.63–0.93) in the institutional test dataset. A similar performance was observed in the external test dataset with a median F1-score of 1.0 (IQR = 0.89–1.0), a median L-sensitivity of 0.85 (IQR = 0.76–0.94) and a median L-PPV of 0.76 (IQR = 0.68–0.88).

### Correlation Between Ground-Truth and Predicted Volumes by HD-BM

**Figure 4.**
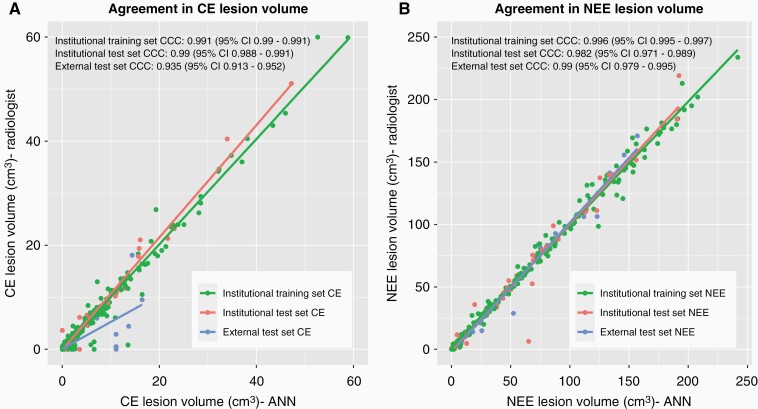
Volumetric agreement between the ground-truth segmentation mask generated by the radiologist and the automatically generated segmentation masks for contrast-enhancing (CE) tumor (A) and non-enhancing FLAIR signal abnormality/edema (NEE) (B).

HD-BM exhibits a strong correlation between the individual CE-lesion volumes (i.e. on a lesion-wise basis) derived from ground-truth segmentation masks vs. those predicted by the HD-BM algorithm in the institutional test dataset (CCC = 0.990 [95% CI = 0.988–0.991]) as well as in the external test dataset (CCC = 0.935 [95% CI = 0.913–0.952]) (Figure 4). Similar performance metrics were obtained when analyzing the NEE volumes (on a case-wise basis) derived from ground-truth segmentation masks vs. those predicted by the HD-BM algorithm in the institutional test dataset (CCC = 0.982 [95% CI = 0.971–0.989]) as well as in the external test dataset (CCC = 0.99 [95% CI = 0.979–0.995]) (Figure 4).

### Full vs. Slim Configuration

Our Slim configuration of HD-BM performs slightly worse across the key metrics, as is to be expected with fewer information available. A detailed discussion and interpretation is provided in the Supplement S4.

### Public Implementation of HD-BM

A public implementation of HD-BM is provided as open-source through www.github.com/NeuroAI-HD/HD-BM.

## Discussion

The application of AI for automatic image processing in neuro-oncology has shown enormous potential to improve the diagnostic and therapeutic decision-making processes.^8,33^ In this paper, we created HD-BM, an ANN-based algorithm for automated volumetric quantification of BM and evaluated its performance in two test datasets: the institutional test dataset with *n* = 62 MRIs of patients with BM (*n* = 384) from different primary malignancies (*n* = 8) and the external test dataset with 30 patients with BM (*n* = 155) from lung cancer. HD-BM performed well for both automated detection and volumetric quantification of BM with high agreement to the radiologist-annotated ground-truth and simultaneously obtaining ≤ 1 FP/scan. In contrast to previous studies HD-BM did not only focus on CE lesions but allowed precise differentiation between CE lesions and the surrounding NEE parts of BM, which may be particularly important in the context of post-treatment changes during follow-up of BM.^4^ Moreover, we showed no need for applying the volumetric threshold to maximize lesion detection performance in contrast to prior studies (most ranging 0.003–4 cm^3^),^19–23^ thereby highlighting the robustness of HD-BM even for small lesions.

A direct comparison of the performance of an algorithm with other works was only possible to a limited extent due to the different underlying data and metrics. HD-BM achieved a high F1-score (> 0.93 in both test sets); previous studies reported F1-scores of 0.76–0.85.^19,21,23^ This can be attributed to the fact that in our analysis a DICE-score of > 0.1 sufficed to be considered a true positive in conjunction with the fact that our method had high detection performance even for small volume lesions. Moreover, our method presented a lower number of FP/scan (0.87 in the institutional and 0.2 in the external test dataset) compared to about 1.5–20 FP/scan in the literature.^17,20,25,27^ While Bousabarah et al.^19^ have shown that smaller BM increased the likelihood of FP, our method achieved a good performance despite the small size of the BM without resulting in more FP or lower sensitivity. We obtained good L-Sensitivity in detecting BM in both test sets (0.81 and 0.85 in institutional and independent test sets respectively), which is well in line with previous studies reporting sensitivities of 0.70–0.96.^17,19,20,22–27^ Zhang et al.^27^ achieved the highest sensitivity of 0.96, but presented more than twenty times the number of FP/scan than HD-BM. This also applied to other works with higher sensitivity, which however also featured about eight times (7.8 FPs/scan)^20^ and two times (1.5 FPs/scan)^25^ more FPs/scan than HD-BM. A recent study by Park et al.^26^ reported a high sensitivity of 0.931 and also low FP/scan with 0.59. They developed multiple methods, the best using a combination of 3D black blood and 3D gradient echo (GRE) imaging techniques, while their model based only the 3D GRE sequences (like ours), reached a sensitivity of 0.768, which is slightly lower than the L-Sensitivity in our test sets.

HD-BM exhibited both high detection performance and few FPs/cases, despite the challenging dataset containing multiple low volume lesions, and performed well on our independent test dataset, indicating high robustness and potential generalizability of our method. We are also confident that HD-BM can be transferred to clinical conditions since the algorithm performed well on heterogeneous data with a broad appearance of BM on MRI including complex post-treatment alterations, like post-operative bleeding. The L-DICE segmentation performance of our HD-BM algorithm (0.78 or 0.79 in both test sets) was in line with previous studies (0.6–0.82).^17,19–23,26^ As expected, on a case-by-case basis our approach showed a better result with a median C-DICE-score of 0.9 in the institutional test set, which is comparable to the results in larger primary brain tumors.^8^ We observed comparatively lower L-DICE as compared to C-DICE values, which can be expected because many patients have multiple lesions of different volumes: When calculating the C-DICE, the L-DICE of the bigger lesions influenced the metric more than smaller lesions, due to the greater number of true positive/false negative/FP voxels of the large lesions. Furthermore, the L-DICE of low volume lesions tends to be lower since the ratio of border voxels to internal voxels increases, leading to a more difficult segmentation problem. Additionally, the L-DICE of low volume lesions tends to be lower as shown in Bousabarah et al.^19^

Our study has some limitations. First, we acknowledge the retrospective design of the study. Although HD-BM performed well on both internal and external test sets, further multicentric validation and refinement may be required to enable future clinical applicability, in order to verify its generalizability to images from different scanners and vendors. In this context, it will also be required to specifically evaluate the performance of HD-BM for longitudinal tracking of BM and response assessment in individual patients. Second, HD-BM required multiparametric MRI data, thus limiting the applicability of our method if one of the four required sequences are missing. To mitigate this, previous studies have shown that missing MRI sequences may be synthesized using generative adversarial networks.^34,35^ Consequently, this may enable the use of HD-BM even with incomplete and heterogeneous sequence protocols.

In conclusion, our results highlight the capability of ANN for reliable detection and precise volumetric quantification of CE and NEE compartments in patients with BM, thereby supporting the assessment of BM disease burden and progression. A public implementation of HD-BM is available through www.github.com/NeuroAI-HD/HD-BM.

## Supplementary Material

vdac138_suppl_Supplementary_Materials

## References

[1] Gavrilovic IT , Posner JB. Brain metastases: epidemiology and pathophysiology. J Neurooncol. 2005;75(1):5–14. 16215811 10.1007/s11060-004-8093-6

[2] Barnholtz-Sloan JS , Yu C, Sloan AE, et al A nomogram for individualized estimation of survival among patients with brain metastasis. Neuro-oncology. 2012;14(7):910–918. 22544733 10.1093/neuonc/nos087 PMC3379797

[3] Lagerwaard FJ , Levendag PC, Nowak PJ, et al Identification of prognostic factors in patients with brain metastases: a review of 1292 patients. Int J Radiat Oncol Biol Phys. 1999;43(4):795–803. 10098435 10.1016/s0360-3016(98)00442-8

[4] Lin NU , Lee EQ, Aoyama H, et al Response assessment criteria for brain metastases: proposal from the RANO group. Lancet Oncol. 2015;16(6):e270–e278. 26065612 10.1016/S1470-2045(15)70057-4

[5] Chow DS , Qi J, Guo X, et al Semiautomated volumetric measurement on postcontrast MR imaging for analysis of recurrent and residual disease in glioblastoma multiforme. Am J Neuroradiol. 2014;35(3):498–503. 23988756 10.3174/ajnr.A3724 PMC7964732

[6] Gahrmann R , van den Bent M, van der Holt B, et al Comparison of 2D (RANO) and volumetric methods for assessment of recurrent glioblastoma treated with bevacizumab—a report from the BELOB trial. Neuro-oncology. 2017;19(6):853–861. 28204639 10.1093/neuonc/now311 PMC5464446

[7] Bauknecht HC , Klingebiel R, Hein P, et al Effect of MRI-based semiautomatic size-assessment in cerebral metastases on the RANO-BM classification. Clin Neuroradiol. 2020;30(2):263–270. 31197388 10.1007/s00062-019-00785-1

[8] Kickingereder P , Isensee F, Tursunova I, et al Automated quantitative tumour response assessment of MRI in neuro-oncology with artificial neural networks: a multicentre, retrospective study. Lancet Oncol. 2019;20(5):728–740. 30952559 10.1016/S1470-2045(19)30098-1

[9] Chang K , Beers AL, Bai HX, et al Automatic assessment of glioma burden: a deep learning algorithm for fully automated volumetric and bidimensional measurement. Neuro-oncology. 2019;21(11):1412–1422. 31190077 10.1093/neuonc/noz106 PMC6827825

[10] Ali MJ , Raza B, Shahid AR. Multi-level Kronecker Convolutional Neural Network (ML-KCNN) for glioma segmentation from multi-modal MRI volumetric data. J Digit Imaging. 2021;34(4):905–921. 34327627 10.1007/s10278-021-00486-7 PMC8455792

[11] Bouget D , Eijgelaar RS, Pedersen A, et al Glioblastoma surgery imaging-reporting and data system: validation and performance of the automated segmentation task. Cancers. 2021;13(18):4674. 34572900 10.3390/cancers13184674 PMC8465753

[12] Di Ieva A , Russo C, Liu S, et al Application of deep learning for automatic segmentation of brain tumors on magnetic resonance imaging: a heuristic approach in the clinical scenario. Neuroradiology. 2021;63(8):1253–1262. 33501512 10.1007/s00234-021-02649-3

[13] Ermiş E , Jungo A, Poel R, et al Fully automated brain resection cavity delineation for radiation target volume definition in glioblastoma patients using deep learning. Radiat Oncol. 2020;15(1):1–10. 10.1186/s13014-020-01553-z PMC7204033 32375839

[14] Rudie JD , Weiss DA, Saluja R, et al Multi-disease segmentation of gliomas and white matter hyperintensities in the BraTS data using a 3D convolutional neural network. Front Comput Neurosci. 2019;13:84. 31920609 10.3389/fncom.2019.00084 PMC6933520

[15] Tampu IE , Haj-Hosseini N, Eklund A. Does anatomical contextual information improve 3D U-Net-based brain tumor segmentation? Diagnostics (Basel, Switzerland). 2021;11(7):1–15. 10.3390/diagnostics11071159 PMC8306843 34201964

[16] Zadeh Shirazi A , McDonnell MD, Fornaciari E, et al A deep convolutional neural network for segmentation of whole-slide pathology images identifies novel tumour cell-perivascular niche interactions that are associated with poor survival in glioblastoma. Br J Cancer. 2021;125(3):337–350. 33927352 10.1038/s41416-021-01394-x PMC8329064

[17] Grøvik E , Yi D, Iv M, et al Deep learning enables automatic detection and segmentation of brain metastases on multisequence MRI. J Magn Reson Imaging. 2020;51(1):175–182. 31050074 10.1002/jmri.26766 PMC7199496

[18] Yang Z , Liu H, Liu Y, et al A web-based brain metastases segmentation and labeling platform for stereotactic radiosurgery. Med Phys. 2020;47(8):3263–3276. 32333797 10.1002/mp.14201 PMC7567132

[19] Bousabarah K , Ruge M, Brand JS, et al Deep convolutional neural networks for automated segmentation of brain metastases trained on clinical data. Radiat Oncol (Lond, Engl). 2020;15(1):1–9. 10.1186/s13014-020-01514-6 PMC7171921 32312276

[20] Charron O , Lallement A, Jarnet D, et al Automatic detection and segmentation of brain metastases on multimodal MR images with a deep convolutional neural network. Comput Biol Med. 2018;95:43–54. 29455079 10.1016/j.compbiomed.2018.02.004

[21] Pennig L , Shahzad R, Caldeira L, et al Automated detection and segmentation of brain metastases in malignant melanoma: evaluation of a dedicated deep learning model. Am J Neuroradiol. 2021;42(4):655–662. 33541907 10.3174/ajnr.A6982 PMC8040988

[22] Rudie JD , Weiss DA, Colby JB, et al Three-dimensional U-Net convolutional neural network for detection and segmentation of intracranial metastases. Radiol Artif Intell. 2021;3(3):e200204. 34136817 10.1148/ryai.2021200204 PMC8204134

[23] Jünger ST , Hoyer UCI, Schaufler D, et al Fully automated MR detection and segmentation of brain metastases in non-small cell lung cancer using deep learning. J Magn Reson Imaging. 2021;54(5):1608–1622. 34032344 10.1002/jmri.27741

[24] Zhou Z , Sanders JW, Johnson JM, et al Computer-aided detection of brain metastases in T1-weighted MRI for stereotactic radiosurgery using deep learning single-shot detectors. Radiology. 2020;295(2):407–415. 32181729 10.1148/radiol.2020191479 PMC8287889

[25] Kikuchi Y , Togao O, Kikuchi K, et al A deep convolutional neural network-based automatic detection of brain metastases with and without blood vessel suppression. Eur Radiol. 2022;32(5):2998–3005. 34993572 10.1007/s00330-021-08427-2

[26] Park YW , Jun Y, Lee Y, et al Robust performance of deep learning for automatic detection and segmentation of brain metastases using three-dimensional black-blood and three-dimensional gradient echo imaging. Eur Radiol. 2021;31(9):6686–6695. 33738598 10.1007/s00330-021-07783-3

[27] Zhang M , Young GS, Chen H, et al Deep-learning detection of cancer metastases to the brain on MRI. J Magn Reson Imaging. 2020;52(4):1227–1236. 32167652 10.1002/jmri.27129 PMC7487020

[28] Liu Y , Stojadinovic S, Hrycushko B, et al A deep convolutional neural network-based automatic delineation strategy for multiple brain metastases stereotactic radiosurgery. PLoS One. 2017;12(10):e0185844. 28985229 10.1371/journal.pone.0185844 PMC5630188

[29] Isensee F , Jaeger PF, Kohl SAA, Petersen J, Maier-Hein KH. nnU-Net: a self-configuring method for deep learning-based biomedical image segmentation. Nat Methods. 2021;18(2):203–211. 33288961 10.1038/s41592-020-01008-z

[30] Isensee F , Schell M, Pflueger I, et al Automated brain extraction of multisequence MRI using artificial neural networks. Hum Brain Mapp. 2019;40(17):4952–4964. 31403237 10.1002/hbm.24750 PMC6865732

[31] Isensee F , Jäger PF, Full PM, Vollmuth P, Maier-Hein KH.nnU-Net for brain tumor segmentation. In: Crimi A, Bakas S, eds. Brainlesion: Glioma, Multiple Sclerosis, Stroke and Traumatic Brain Injuries. BrainLes 2020. Lecture Notes in Computer Science, vol 12659. Springer, Cham; 2021. doi:10.1007/978-3-030-72087-2_11

[32] Ronneberger O , Fischer P, Brox T. U-Net: convolutional networks for biomedical image segmentation. In: Navab N, Hornegger J, Wells W, Frangi A, eds. Medical Image Computing and Computer-Assisted Intervention – MICCAI 2015. MICCAI 2015. Lecture Notes in Computer Science, vol 9351. Springer, Cham; 2015. doi:10.1007/978-3-319-24574-4_28

[33] Laukamp KR , Pennig L, Thiele F, et al Automated meningioma segmentation in multiparametric MRI. Clin Neuroradiol. 2021;31(2):357–366. 32060575 10.1007/s00062-020-00884-4

[34] Conte GM , Weston AD, Vogelsang DC, et al Generative adversarial networks to synthesize missing T1 and FLAIR MRI sequences for use in a multisequence brain tumor segmentation model. Radiology. 2021;299(2):313–323. 33687284 10.1148/radiol.2021203786 PMC8111364

[35] Jayachandran Preetha C , Meredig H, Brugnara G, et al Deep-learning-based synthesis of post-contrast T1-weighted MRI for tumour response assessment in neuro-oncology: a multicentre, retrospective cohort study. Lancet Digit Health. 2021;3(12):e784–e794. 34688602 10.1016/S2589-7500(21)00205-3

