# Supplementary material for: Automated detection and quantification of brain metastases on clinical MRI data using artificial neural networks
Source: Neurooncol Adv. 2022 Aug 23;4(1):vdac138. doi: 10.1093/noajnl/vdac138 (PMC9466273; doi:10.1093/noajnl/vdac138)
Supplement: vdac138_suppl_Supplementary_Materials [file vdac138_suppl_supplementary_materials.docx]

**Supplementary Materials**

**Supplementary Methods S1: Overview of used MRI Sequences and acquisition parameters**

3T MRI: Sagittal T1-weighted 3D MPRAGE images were acquired both before and after administration of a 0.1 mmol/kg dose of gadoterate meglumine (DOTAREM) with the following parameters: TE=3.7 ms, TR=1790 ms and Flip angle (FA)=15°, in-plane resolution 0.78x0.78x1.0 mm, section thickness 1 mm. Axial T1-weighted flash 2D images were acquired with TE=4.75 ms, TR=301 ms, in-plane resolution 0.6x0.6x5.0 mm, FA=90° and section thickness = 5 mm and an interslice gap of 10%. Axial FLAIR images were acquired with TE=136 ms, TR=8500 ms, in-plane resolution 0.9x0.9x5.0 mm, FA=170° and section thickness = 5 mm and an interslice gap of 10%.

1.5T MRI: Axial T1-weighted 3D MPRAGE images both before and after administration of a 0.1 mmol/kg dose of gadobutrol (Gadovist) with TE=3.02 ms, TR=2000 ms, in-plane resolution 1x1x1 mm, section thickness 1mm and FA=8°. Axial FLAIR images were acquired with TE=121 ms, TR=8500 ms, in-plane resolution 0.7x0.7x5 mm, section thickness = 5 mm, FA=180° and an interslice gap of 10%.

T2-weighted images had a different orientation in independent (coronal) and institutional (axial) datasets, therefore these were not used for the ANN development.

**Supplementary Methods S2: Artificial Neural Network (ANN)**

In this section we provide further details on our developed ANN for automated brain metastasis segmentation based on the popular state-of-the-art method nnU-Net,^1^ which in turn is based on the well-established U-Net architecture^2^ (implemented with Python 3.9.7 (www.python.org) using the PyTorch^3^ package version 1.9.1 (www.pytorch.org) and computed on RTX 2080 TI GPUs from NVIDIA).

*Network input preprocessing*

Due to varying spacings between cases, we resampled the original data of the scans to a common spacing of 1.0 mm x 0.78125 mm x 0.78125 mm [Z x Y x X], in order to expose our model to a consistent geometry. Following this, a per-patient, per-modality intensity normalisation was conducted by subtracting the mean intensity value and dividing by the standard deviation. Intensity normalisation was applied only to the brain region in the images. The non-brain voxels remained 0. Finally, images are cropped to the brain voxels by removing the zero-valued borders.

*Network Architecture*

The network architecture we use is a derivative of the well-established 3D U-Net architecture first introduced by Ronneberger et al.^2,4^ which was automatically configured by the nnU-Net framework.^1^ The U-Net architecture consists of blocks of convolutional, instance normalization, and nonlinearity layers, which get downsampled iteratively in an encoder stage. This allows the network to aggregate global semantic information by reducing the spatial extent. Once the lowest resolution is reached the decoder reconstructs the spatial information through a combination of the global semantic information and lateral skip connections which are directly passed from the encoder to the decoder stage of the same spatial resolution. In a nutshell this reconstruction allows the decoder to more accurately localise structures of interest in the image.

Fig. S1 illustrated the exact U-Net architecture including the number of channels, spatial resolution and kernels. The architecture is fully convolutional, featuring convolution kernels of size 3x3x3, downsampling is done via strided convolutions and upsampling is achieved through transposed convolutions.

*Training Procedure*

During training, we crop patches of the preprocessed MRI scans to size 112x128x160, as automatically configured by nnUNet, and aggregate two of these crops to mini-batches, which are subsequently fed into the ANN. We optimise our network from scratch using SGD with Nesterov momentum (initial learning rate (LR) 0.1, momentum 0.99, weight decay 3e-05) for T=1000 epochs and 250 mini-batches per epoch. The LR gradually decreases with growing epochs, according to the PolyLR learning rate schedule: $LR\left( t \right)={LR}_{init}\left( \frac{\left( 1-t \right)}{T} \right)$, where t denotes the current epoch. As loss we employ an equally weighted sum of a soft dice loss^5,6^ and cross entropy loss in combination with deep supervision, where we predict segmentation maps at the 4 biggest spatial resolutions in order to improve gradient propagation and calculate a weighted sum of these losses with weights (0.53, 0.27, 0.13, 0.07 – from large spatial resolution to small resolution).

*Data Augmentation*

In order to prevent overfitting of HD-BM and to improve its robustness to aberrations we chose to heavily augment our data on the fly utilising our in-house developed augmentation library BatchGenerators^7^ v0.21 (available at https://github.com/MIC-DKFZ/batchgenerators). In the following augmentation scheme we denote uniformly drawn values between a range x and y as U[x, y], denote the probability of an augmentation getting applied to different input modalities (channels) independently as p_ch_ and denote the probability an augmentations gets applied to an independent axis as p_ax_. Our augmentation pipeline significantly extends the default nnU-Net augmentation pipeline, especially the novel local intensity transforms that increase the diversity of our patches by a large margin. This lead to the following schedule:

1. 40% chance of rotating the patches around the axes at random angles U[-30°,30°] and p_ax_=50%
2. 20% chance of scaling the patches independently along all three axis by a random factor of U[0.7, 1.43] and p_ax_=100%
3. Randomly choosing one of the transforms with equal probability:
   1. 20% chance of applying a Median Filter of size U[2, 8] and p_ch_=50%
   2. 20% chance of applying a Gaussian Blur with 𝜎=U[0.3, 1.5] and p_ch_=50%
4. 10% chance of adding Gaussian Noise with 𝜎=U[0, 0.1] and p_ch_=100%
5. 10% chance of augmenting the Brightness with 𝜇=0, 𝜎=0.5 and p_ch_=50%
6. Randomly choosing one of the transforms with equal probability:
   1. 20% chance of reducing/increasing the contrast by U[0.5, 2] with p_ch_=50%
   2. 20% chance of changing the contrast as above but clipping the values when exceeding the original intensity range
7. 15% chance of introducing down/upsampling artefacts by consecutive linear down and upsampling with scale ranges between U[0.25, 1]
8. Two times 10% chance of changing Gamma with $I'=I^{\gamma}with\gamma\in U\left[ 0.7,1.5 \right]$ and scaling the result to maintain original mean and std
9. 50% chance of mirroring each axis
10. 40% chance of blacking out $n=U\left[ 1,5 \right]$3D patches of dimensions d_1_=U[11,37], d_2_=U[12, 42], d_3_=U[16, 53] for the respective axis and p_ch_=50%
11. 30% chance of adding gaussian intensity gradients similar to bias fields with p_ch_=50% of scale s_1_=exp(U[log(18), log(112)]), s_2_=exp(U[log(21), log(128)]), s_3_=exp(U[log(26), log(160)]) and either strength t=U[-5,-1] or t=U[1, 5].
12. 30% chance of adding local gamma transforms with p_ch_=50% and of scale s_1_=exp(U[log(18), log(112)]), s_2_=exp(U[log(21), log(128)]), s_3_=exp(U[log(26), log(160)]) as well as max strength t=U[0.01, 0.8] or t=U[1.5, 4]
13. 20% chance of applying a sharpening transform with strength U[0.1, 1] with p_ch_=50%

*Training and evaluation procedure*

We leveraged the institutional training dataset to train and validate 5 different ANNs by partitioning the dataset into 5 disjoint folds, each comprising 20% of the samples. Each ANN is trained from scratch on the remaining 80% (all samples but its 20% fold) of the training data and validates its performance on it. Since we trained on smaller patches, but want to predict the entire images, we employed a sliding window approach with half patch size overlap between predictions in each dimension. Additionally, we wanted to assure the input images are as close as possible to the images we exposed our method to during training, therefore we employed the same preprocessing steps as described in the main body: We resampled the images to the same spacing 1.0 mm x 0.78125 mm x 0.78125 mm and we normalised the MRI intensity values as described above. Given the preprocessed images we investigated whether test-time augmentations improved the performance on the validation dataset but found no benefit in using it, hence we omitted it.

For predicting our institutional and independent test sets we used the five models obtained from the cross-validation on the training dataset as an ensemble. Specifically, we predicted the test cases with each model independently, then averaged their softmax outputs for the final prediction. These final predictions are resampled with trilinear interpolation to the resolution of the original MRI sequence, converted to segmentation maps through the argmax operator and subsequently used as source for the case and lesion wise evaluation.

**Supplementary Methods S3: ANN evaluation metrics**

Our evaluation metrics can be divided in two sub-sections: The case-wise evaluation where we focused on the performance on a whole case without considering tumor instances and the lesion-wise evaluation where we evaluated how well our ANN performs over the tumor instances of our cohort. The Shapiro–Wilk test was performed to compare all evaluation metrics for normality. Group differences between all datasets were evaluated with chi-square test for categorical and Kruskal-Wallis test or *t* test (depending on the distribution) for continuous parameters. The Wilcoxon test was performed to determine a statistical difference between the institutional training/test and external test set dataset. Statistical analysis was performed using R (R version 4.0.3, R Foundation for Statistical Computing) as well as Python (Python Software Foundation, version 3.9.7, http://www.python.org).

*Case-wise evaluation:*

For our case-wise evaluation we utilised our trained HD-BM method to predict the cases after preprocessing them as we did for our training. Based on these predictions we then determined if predicted voxels are correct w.r.t. the GT resulting in voxels which are either true positive (TP) or false negative (FP). False negatives (FN) are groundtruth voxels which were not correctly predicted by the method. These can subsequently be used to calculate the case-wise DICE similarity coefficients (C-DICE), case-wise sensitivity (C-Sensitivity) and the concordance correlation coefficient (CCC) with the formulas given below.

*Lesion-wise evaluation:*

To be able to create a lesion-wise evaluation we had to create lesion instances through a morphological analysis. To conduct this analysis, we resampled the predictions and ground truths to an isotropic spacing of 1x1x1 mm. Given the same spacing along all axes we then applied a morphological dilation using the morphology module of scikit-image (v0.18.1) with a ball of radius r=3 to connect voxels that were in close proximity to each other. After the dilation we ran a connected components analysis with a 3D-Ball kernel to determine which voxels belonged to which instances. To determine the optimal size of the 3D-Ball and the dilation we evaluated a broad sweep of different configurations on 10 cases of the training set and visually validated the quality of the instance creation. Given these newly created instances we calculated the lesion instance segmentation agreement using DICE (L-DICE) between each GT and each predicted (PD) instance of the CE tumor class. To measure the detection performance of HD-BM we considered a whole PD lesion instance a TP when it matched any GT lesion instance with L-DICE ≥ 0.1 and a FP else. When a GT lesion instance was not matched by any PD lesion instance with L-DICE ≥ 0.1 it was considered a FN, true negatives don’t exist in the lesion instance-based evaluation. This allowed us to calculate the detection metrics of the lesion-wise sensitivity (L-Sensitivity), the lesion-wise positive predictive value (L-PPV), the lesion-wise F1-Score (L-F1) and concordance correlation coefficient metrics.

We calculated the lesion-wise metrics for each case to reduce the impact few cases with many instances would have on the overall performance. To highlight this issue consider the following example: Our institutional test-dataset consists of 62 patients and 384 CE instances in total. Two patients contribute 118 instances, which is about 30% of all instances. If we calculated the F1-score without taking the case the lesion originates from into account 30% of our HD-BM performance would be dependent on HD-BMs performance on these two patients. By taking the cases into account we put more emphasis on the performance of each patient instead of cases with more lesions. On the other hand, calculating the L-F1 metric this way comes with some problems: 18 of our 62 cases in the institutional test set have a single BM instance. This makes for very ‘noisy’ L-F1-scores since, if HD-BM does not detect the tumor in such an instance the L-F1 for this case will be zero, and if it detects it (without another FP) we have an L-F1 of 1.0, leading to highly volatile results in cases with a low-number of BMs. We therefore report median due to the non-normality of our distribution but also opt to report the mean to provide more information of HD-BMs performance.

We provide a short description and formulas of the previously mentioned metrics:

- C-Sensitivity is defined as the ratio of the overlap volume between prediction mask (PM) and ground truth mask (GT) to the volume of the GT.

$$Sensitivity= \frac{|GT\cap PM|}{\left| GT \right|}$$

- L-Sensitivity is defined as the ratio of true positive lesion instances to the actual positives [true positive + false negative].

$$Sensitivity=\frac{true positive}{true positive+false negative}$$

- L-PPV is defined as the ratio of true positive lesion instances to the sum of true positive and FP.

$$PPV=\frac{true positive}{true positive+FP}$$

- F1-score score combines both information of sensitivity and specificity into one metric and can take values between 0 and 1, where 1 indicates perfect performance. We calculate the F1 score for each case on a lesion-wise basis and average it.

$$F1-score=\frac{2true positive}{2true positive+false negative+FP}$$

- The DICE coefficient is a standard metric for reporting the performance of segmentation tasks, it relates the total areas of the PM and GT to the area of their overlap (GT$\cap$PM). The DICE score can take values between 0 (no overlap between PM and GT) and 1 (PM is identical to GT).

$$DICE= \frac{2 |GT\cap PM|}{\left| GT \right|+|PM|}$$

- We use the CCC to measure how well correlated the volumes of the ANN predictions are to the actual volumes provided by the GT, without taking their overlap into account. In the case of perfect concordance we would get a CCC of 1 (when all samples would populate the 45 degree line in the scatter plots).

**Supplementary Methods S4: Slim MRI configuration**

Our developed full method requires four input modalities: T1-weighted images before gadolinium contrast (T1w), T1-weighted images after gadolinium contrast (T1ce), FLAIR images and a T1-subtraction maps between the T1-weighted images before and after gadolinium contrast (T1-sub). Including all three imaging sequences needed for our inputs in clinical practice can incur more time and costs and furthermore constraints the usability of our method. Therefore we evaluated how our method behaves when trained with only a "Slim" version of the input sequences. Specifically we omit the T1w sequence and consequently the T1sub sequence, which depends on the T1w image in its creation process. As highlighted in the main body we observe that our method loses performance slightly. For CE-Lesions the median case-wise C-DICE drops from 0.90 (0.85 - 0.94) to 0.89 (0.81 - 0.93) and drops in C-Sensitivity from originally 0.91 (0.82-0.95) to 0.89 (0.80 - 0.95) on the internal test set in the Slim version. Both of these decreases are statistically significant. In the instance wise evaluation of CE-Lesions we observe similar behavior: For the L-DICE we observe a decrease from 0.78 (0.60 - 0.91) to 0.71 (0.53 - 0.88) and for the L-Sensitivity a reduction from 0.81 (0.63 - 0.92) to 0.72 (0.54 - 0.91). For L-PPV and the F1-Score we don't observe significant changes from the original values. We observe a largely similar behavior for the CE-Lesions in the external test set, depite some values being non-significant as the sample size is a lot smaller in the external test set. For a comprehensive list of all specific metric- and p-values we refer to Table 2.

We hypothesize that the model lacks context information due to the absence of the T1w and T1-sub sequence, leading to a more difficult problem in delineating CE-lesions and resulting in decreased C-DICE and L-DICE.

Regarding the NEE-Lesions we observed that the C-DICE increases slightly from 0.85 (0.72-0.91) to 0.86 (0.72-0.91) in external test set while does not change from its original values 0.96 (0.92 - 0.97) in institutional test set. We observed similar behavior for the C-Sensitivity in both test sets, despite some values being non-significant. We expected the model to largely stay the same since most information regarding NEE-Lesions is contained in the FLAIR sequence, which is still part of the model inputs. This is also what we observe in the results since most changes are non-significant changes and deviate only slightly from the original values.

All in all we observe that even without T1w and T1-sub modalities our model is able to predict CE- and NEE-Lesions accurately. The C-DICE values decreased slightly for the independent and the external test sets but is still of high quality with median C-DICE values of 0.89 and 0.83 for the internal and external test set. For NEE-Lesions we observe hardly a change in performance, and even a slight increase in the median C-DICE of the external test set. Regarding the lesion-wise performance for CE-lesions we observe that the F1-Score on the test sets does not change significantly, indicating that BM instances can still be found and detection quality is maintained, however the average delineation quality decreases as indicated by L-DICE and L-Sensitivity changes.”

**Supplementary References**

**1.** Isensee F, Jaeger PF, Kohl SAA, Petersen J, Maier-Hein KH. nnU-Net: a self-configuring method for deep learning-based biomedical image segmentation. *Nature Methods.* 2021; 18(2):203-211.

**2.** Ronneberger O, Fischer P, Brox T. U-Net: Convolutional Networks for Biomedical Image Segmentation2015; Cham.

**3.** Paszke A, Gross S, Massa F, et al. Pytorch: An imperative style, high-performance deep learning library. *J Advances in neural information processing systems.* 2019; 32:8024–8035.

**4.** Çiçek Ö, Abdulkadir A, Lienkamp SS, Brox T, Ronneberger O. 3D U-Net: Learning Dense Volumetric Segmentation from Sparse Annotation2016; Cham.

**5.** Drozdzal M, Vorontsov E, Chartrand G, Kadoury S, Pal C. The Importance of Skip Connections in Biomedical Image Segmentation2016; Cham.

**6.** Milletari F, Navab N, Ahmadi S-A. V-net: Fully convolutional neural networks for volumetric medical image segmentation. Paper presented at: 2016 fourth international conference on 3D vision (3DV)2016.

**7.** Isensee F, Jäger P, Wasserthal J, et al. batchgenerators—a python framework for data augmentation. 2020.

**8.** Rudie JD, Weiss DA, Saluja R, et al. Multi-Disease Segmentation of Gliomas and White Matter Hyperintensities in the BraTS Data Using a 3D Convolutional Neural Network. *Frontiers in computational neuroscience.* 2019; 13:84.

**Supplementary Figures**

**
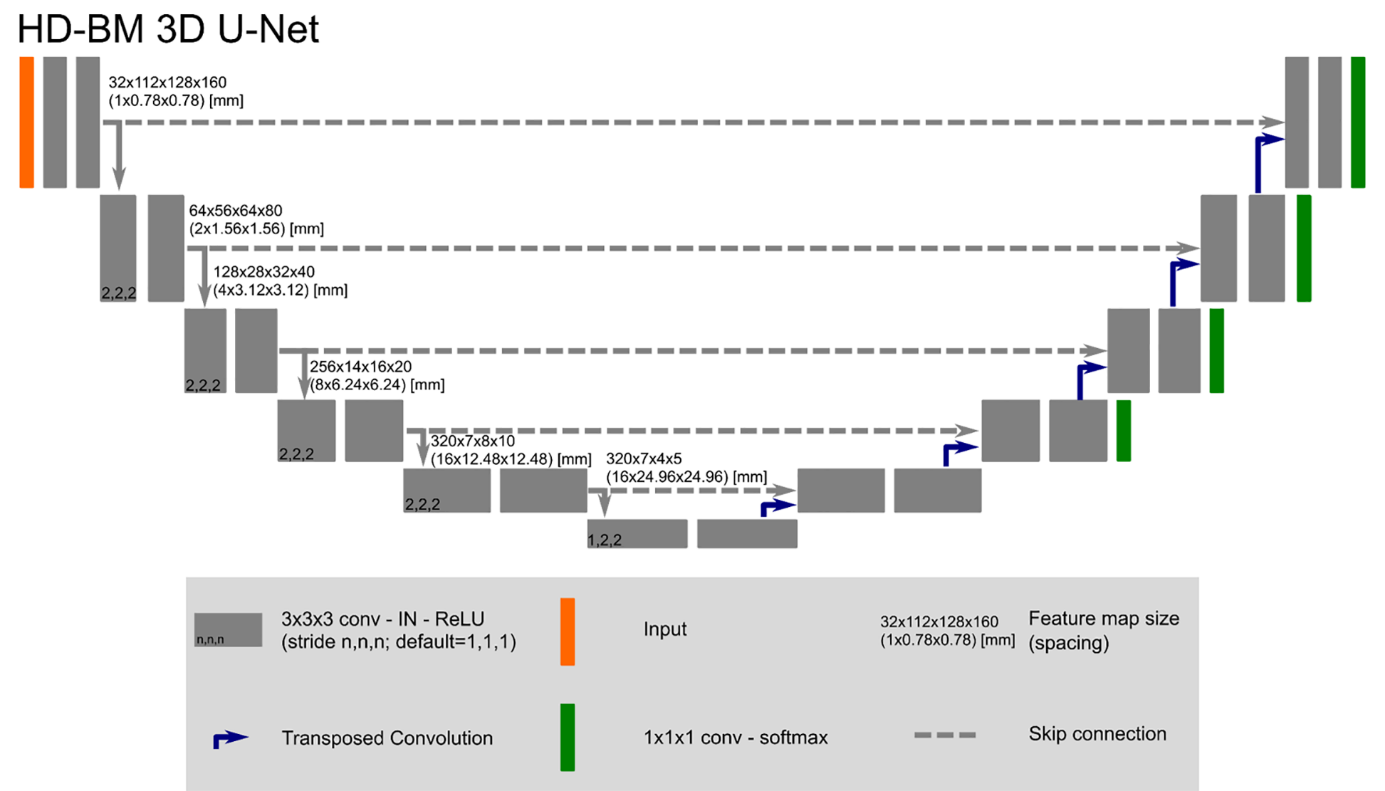
**

**Supplementary Figure S1** Visualisation of the HD-BM architecture for automated Brain Metastasis detection and segmentation. HD-BM follows the U-Net (2) and nnU-Net (1) paradigm of an iterative spatial downsampling followed by upsampling through transposed convolutions and the skip-connections from the encoder to the decoder. It is to note that, due to the different spatial extent we do not downsample all dimensions in each step, as highlighted in the figure.


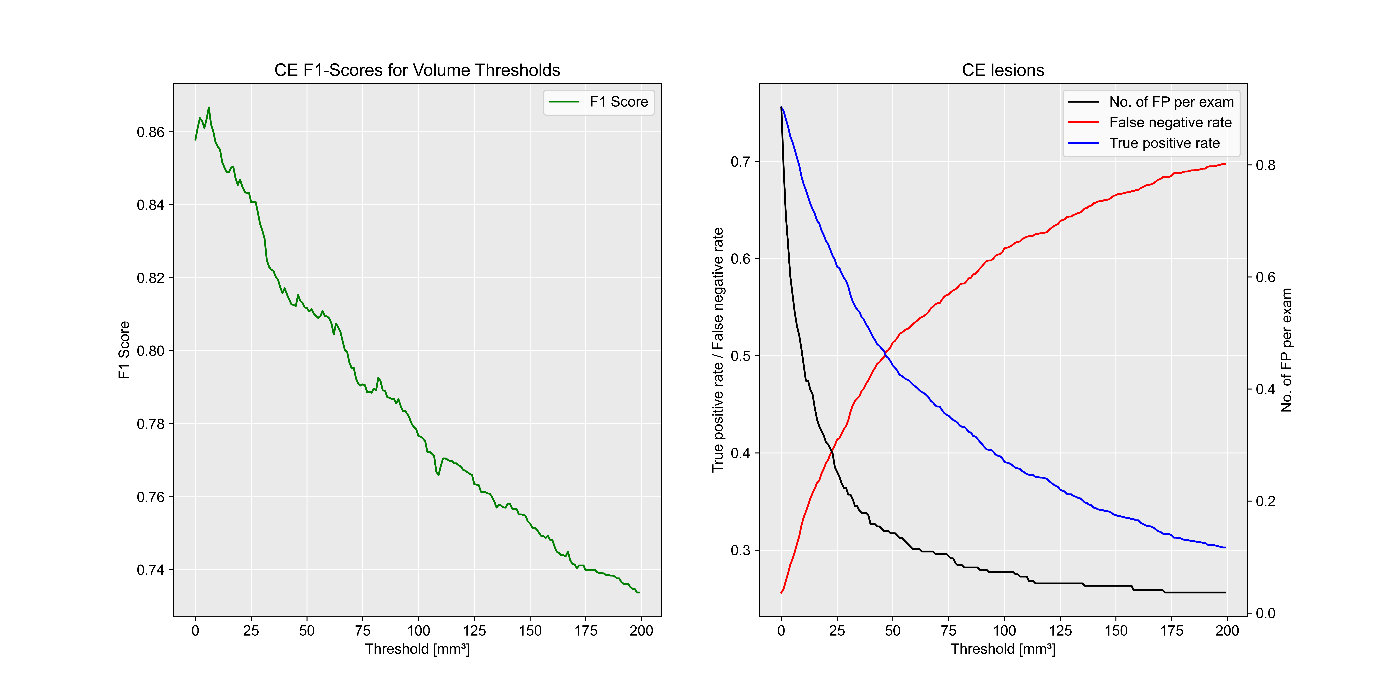


**Supplementary Figure S2:** Illustration of mean F1-score **(left)** as well as true positive rate, false negative rate and No. of false positive (FP) per exam **(right)** for a range of different volumetric thresholds. One can observe a non-significant increase in the F1-score (p=0.2028) when removing low volume lesions, reaching the maximum at 5 mm³. We therefore opted to not apply a volumetric threshold, leading to a lower false negative rate but a higher amount of FP per exam.


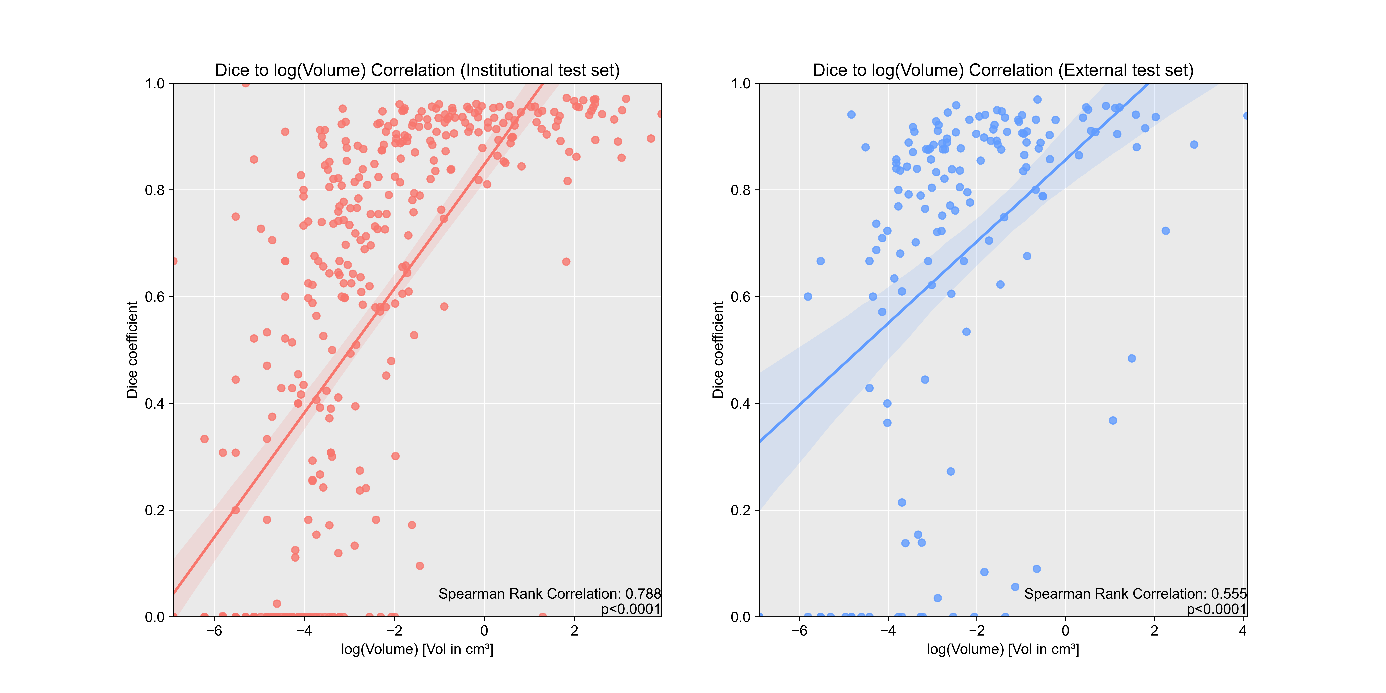


**Supplementary Figure S3:** We observe that HD-BM on average delineates contrast enhancing (CE) lesions better when their volume is high, as shown in ^8^. While the relationship is non-linear the spearman correlation between dice and log of the volume is 0.788 in the institutional and 0.555 in the external test set, both with p<0.0001.
